# A multicentre, randomised, double-blind, single-dose study assessing the efficacy of AMC/DCBA Warm lozenge or AMC/DCBA Cool lozenge in the relief of acute sore throat

**DOI:** 10.1186/1471-2296-12-6

**Published:** 2011-02-18

**Authors:** Alan G Wade, Christopher Morris, Adrian Shephard, Gordon M Crawford, Michael A Goulder

**Affiliations:** 1 CPS Research, Glasgow, UK; 2 Reckitt Benckiser Healthcare International, Hull, UK; 3 Worldwide Clinical Trials, Nottingham, UK

## Abstract

**Background:**

Clinically proven over-the-counter (OTC) treatment options are becoming increasingly important in the self-management of acute sore throat. The aim of this study was to determine the analgesic and sensorial benefits of two different amylmetacresol/2,4-dichlorobenzyl alcohol (AMC/DCBA) throat lozenge formulation variants, AMC/DCBA Warm lozenge and AMC/DCBA Cool lozenge, compared with an unflavoured, non-medicated placebo lozenge in the relief of acute sore throat due to upper respiratory tract infections.

**Methods:**

In this multicentre, randomised, double-blind, single-dose study, 225 adult patients with acute sore throat were randomly assigned to receive either one AMC/DCBA Warm lozenge (n = 77), one AMC/DCBA Cool lozenge (n = 74) or one unflavoured, non-medicated lozenge (matched for size, shape and demulcency; n = 74). After baseline assessments, patients received their assigned lozenge and completed four rating assessments at 11 timepoints from 1 to 120 minutes post dose. Analgesic properties were assessed by comparing severity of throat soreness and sore throat relief ratings. Difficulty in swallowing, throat numbness, functional, sensorial and emotional benefits were also assessed.

**Results:**

Both the AMC/DCBA Warm and AMC/DCBA Cool lozenge induced significant analgesic, functional, sensorial and emotional effects compared with the unflavoured, non-medicated lozenge. Sore throat relief, improvements in throat soreness and difficulty in swallowing, and throat numbness were observed as early as 1-5 minutes, and lasted up to 2 hours post dose. Sensorial benefits of warming and cooling associated with the AMC/DCBA Warm and AMC/DCBA Cool lozenge, respectively, were experienced soon after first dose, and in the case of the latter, it lasted long after the lozenge had dissolved. Emotional benefits of feeling better, happier, less distracted and less frustrated were reported in those taking either of the AMC/DCBA throat lozenge variants, with no differences in adverse events compared with the unflavoured, non-medicated lozenge.

**Conclusions:**

AMC/DCBA Warm and AMC/DCBA Cool lozenges are well-tolerated and effective OTC treatment options, offering functional, sensorial and emotional benefits to patients with acute sore throat, over and above that of the rapid efficacy effects provided.

**Trial registration:**

ISRCTN: ISRCTN00003567

## Background

Acute sore throat is an inflammatory condition characterised by pain, redness, heat and swelling. Inflammatory mediators, such as bradykinin and prostaglandins, released following local responses to cell damage are known to cause some of the sore throat symptoms, including pain and irritation [1]. Pro-inflammatory cytokines have also been linked to 'sickness' behaviour, which in turn is associated with increased pain sensitivity in cases of microbial infection [2]. Moreover, sore throat and 'sickness' are highly correlated, where a greater level of pain experienced by the patient is associated with a greater feeling of sickness/unwellness [3], often resulting in remedial action being sought.

Up to 80% of sore throats are caused by viruses and not bacteria [4,5]. Therefore, antibiotics are ineffective in the majority of cases and are not recommended for the primary treatment of acute sore throat by several clinical bodies in the EU [6,7]. Clinically proven over-the-counter (OTC) options that are rapid-acting, safe and effective in providing symptomatic relief to acute sore throat are becoming increasingly important in the self-management of this condition. Amylmetacresol/2,4-dichlorobenzyl alcohol (AMC/DCBA) throat lozenges (more commonly known as Strepsils^® ^lozenges; Reckitt Benckiser Healthcare International, UK) are a range of medicated throat lozenges available OTC in the UK and many other countries for the symptomatic relief of mouth and throat infections, and acute sore throat. All AMC/DCBA throat lozenges contain the two core active ingredients, AMC (0.6 mg) and DCBA (1.2 mg), both of which possess antibacterial [8,9], antiviral [10] and local anaesthetic [11] properties. Furthermore, clinical evidence available supports the efficacy and safety of AMC/DCBA throat lozenges in the rapid relief of acute sore throat due to upper respiratory tract infections (URTIs) [12-15]. It should be noted that although such OTC products are used to provide symptomatic relief of viral and bacterial pharyngitis, worsening of symptoms may suggest a more serious cause and the opinion of a medical professional should be sought. Data suggest that the analgesic and functional benefits observed with AMC/DCBA throat lozenges were attributable to the active ingredients, AMC and DCBA, as these benefits were significantly over and above the demulcent properties of non-medicated lozenges [15]. AMC/DCBA Cool lozenge is a formulation variant of AMC/DCBA throat lozenge that has been associated with sensorial effects such as cooling, coating and soothing in healthy volunteers [16]. However, up until now neither the efficacy nor the sensorial benefits of this lozenge formulation have been investigated in patients with acute sore throat, hence the basis of part of this study.

In a more recent monadic, in-home, consumer research study conducted by Reckitt Benckiser Healthcare International that adopted the same methodology as Shephard *et al*. [16], approximately 86% of participants said they like to feel 'warm' and comforted when they are suffering from a sore throat (Personal Communications). When these healthy adult volunteers were randomly allocated to one of two lozenges, either a new AMC/DCBA throat lozenge variant (known as AMC/DCBA Warm lozenge) or a control lozenge, more participants taking the AMC/DCBA Warm lozenge (89%) said they felt a warming sensation than those who took the control lozenge (9%; p = 0.01). Sixty-five percent of participants felt it within 30 seconds and 85% felt the effects began in the first minute. More participants taking the AMC/DCBA Warm lozenge (58%) agreed that the experience provided by the Warm lozenge was 'comforting' compared with 23% in the control lozenge group (p < 0.0001). This consumer research study demonstrated that the AMC/DCBA Warm lozenge provided sensorial effects in healthy volunteers, but it is not known whether these effects would be seen in patients with acute sore throat, and what other benefits the product could deliver in this patient population.

This multicentre, randomised, double-blind, single-dose study was designed to examine the analgesic properties of the two AMC/DCBA throat lozenge variants (AMC/DCBA Warm and AMC/DCBA Cool lozenges) compared with an unflavoured, non-medicated placebo lozenge (referred to herein as unflavoured, non-medicated lozenge) in patients with acute sore throat over a period of 2 hours. In addition, sensorial and emotional benefits were investigated via a patient questionnaire survey.

## Methods

### Participants

This study was approved by Fife & Forth Valley Research Ethics Committee. Between 12 January and 20 February 2009, 225 patients with acute sore throat due to URTI who attended a GP referral practice or attended CPS Research in the UK directly in response to advertising were enrolled into the study. Eligible patients were male or female adults aged between 16 and 75, with a confirmed diagnosis of acute sore throat due to URTI and an onset within the previous 4 days. All patients gave written informed consent and had a sore throat score of ≥6 on the Throat Soreness Scale at baseline and the presence of tonsillopharyngitis, as confirmed by the study physicians recording a score of ≥3 points on the expanded 21-point Tonsillopharyngitis Assessment (TPA).

### Interventions

Patients were randomised to receive either one of two AMC/DCBA throat lozenge variants or an unflavoured, non-medicated lozenge (placebo):

i. AMC/DCBA Cool lozenge, containing the active ingredients AMC (0.6 mg) and DCBA (1.2 mg), and various excipients including specially formulated 'cooling flavours'; also known as Strepsils^® ^Cool lozenge; Reckitt Benckiser Healthcare International

ii. AMC/DCBA Warm lozenge, containing the active ingredients AMC (0.6 mg) and DCBA (1.2 mg), and various excipients including specially formulated 'warming flavours'; Reckitt Benckiser Healthcare International

iii. Unflavoured, non-medicated lozenge (sugar-based non-medicated placebo lozenge; size, shape and demulcency matched with the two AMC/DCBA throat lozenge variants)

Note that the placebo lozenge was unflavoured, while the active lozenges were flavoured. The marked difference in flavour between the two active lozenges and the flavour associated with the active ingredients themselves made it impossible to introduce a control specific for this variable.

### Objectives

The primary objective of this study was to determine the analgesic properties of two new formulation variants of AMC/DCBA throat lozenges (AMC/DCBA Cool and AMC/DCBA Warm lozenges) in patients with acute sore throat compared with an unflavoured, non-medicated lozenge.

In addition to the analgesic endpoints, functional measures of difficulty in swallowing and throat numbness were also assessed. Secondary objectives of this study were to determine functional impairment scores, emotional and sensorial benefits of the lozenges via responses to a consumer questionnaire.

### Primary and secondary outcome measures

The primary efficacy endpoint for this study was area under the change-from-baseline curve (AUC) in severity of throat soreness from 0 to 2 hours. The secondary efficacy endpoints were:

• Change from baseline in severity of throat soreness (using the 11-point Throat Soreness Scale); sore throat relief (using a 7-point scale); change from baseline in difficulty in swallowing (using a 100 mm visual analogue scale [VAS] from 'not difficult' to 'very difficult' to swallow); and throat numbness (using a 5-point categorical scale), all measured at 1, 5, 10, 15, 30, 45, 60, 75, 90, 105 and 120 minutes post dose

• Total sum of pain relief ratings (TOTPAR): AUC from baseline to 2 hours post first dosing for sore throat relief

• AUC from baseline to 2 hours for the change from baseline in difficulty swallowing (using a 100 mm visual analogue scale [VAS] from 'not difficult' to 'very difficult' to swallow)

• AUC for throat numbness (using a 5-point scale) measurements from 1 to 120 minutes

• Responses to consumer questionnaire relating to functional impairment and to patients' opinions about the type of pain relief provided; sensorial benefits experienced (i.e. warming and cooling sensations); the speed and duration of effects experienced; emotional benefits, i.e. if they felt any better, any happier, any less frustrated, or any less distracted than before taking a lozenge; and overall treatment rating at 2 hours

### Sample size determination

Sample size determination was based on a previous study with AMC/DCBA throat lozenges conducted at the same research centre [14]. The difference in the mean AUC for the change from baseline in the severity of throat soreness from 0 to 2 hours between the AMC/DCBA throat lozenges and unflavoured, non-medicated lozenges for patients with a TPA ≥3 was assumed to be of similar magnitude and therefore 75 patients per group would be sufficient to provide 90% power to detect a difference of 0.58 in the mean AUC (75% of the effect observed in the previous study) between either of the two test lozenges and the unflavoured, non-medicated lozenge using a 2-tailed two sample *t*-test at the 5% significance level.

### Randomisation and blinding

The method of randomisation used for the assignment of patients to treatment groups was similar to that described previously by McNally *et al*. [15]. A third-party blinding method was employed, where each patient was blindfolded and provided with a single lozenge (from an opaque blister pack) in the clinic by an independent member of the investigational staff who was not involved in the study assessments. Patients were advised that they might experience a cool or warm sensation. Once the lozenge was put into the mouth, the blindfold was removed. Patients were instructed to suck the lozenge slowly, moving the lozenge around the mouth, until it had dissolved, without chewing or crunching the lozenge.

### Assessments

At the screening visit, a medical history, current medical use, therapy history in the past 14 days, baseline TPA and the patient's current medical status were confirmed and recorded by the investigator. At the pre-dosing stage and under supervision of the study nurse or investigator, patients recorded throat soreness and difficulty in swallowing scores. Post dose, patients recorded throat soreness, difficulty in swallowing, throat numbness and sore throat relief scores at 1, 5, 10, 15, 30, 45, 60, 75, 90, 105 and 120 minutes. In addition, patients completed the patient questionnaire pre-dose, and at time intervals of 1, 5, 20, 60 and 120 minutes post dose. All adverse events reported spontaneously by the patient or in response to questioning or observation by the investigator and/or the study nurse pre-dose, 2 hours post dose and at the follow-up visit (1-3 days post dose) were recorded in the patient's case report form.

### Statistical analyses

All statistical tests performed were 2-tailed with significance determined by reference to the 5% significance level, unless otherwise stated. The null hypothesis at all times was the equality of each of the test lozenges being compared with an unflavoured, non-medicated lozenge. Comparisons between each of the test lozenges and unflavoured, non-medicated lozenge were reported with 95% confidence intervals (CIs) for the difference. The primary efficacy endpoint was analysed by analysis of covariance (ANCOVA) with baseline throat soreness severity as a covariate and factors for treatment group and referral centre. Treatment group differences were estimated using the mean square error from the ANCOVA and using Fisher's protected Least Square Difference method.

All secondary endpoints and the supportive analyses were considered as descriptive evidence of efficacy and were analysed without any procedures to account for multiple comparisons. Pair-wise differences between treatment groups in the proportion of patients reporting treatment-emergent adverse events were compared via chi-square tests. Questions with binary responses were analysed using a logistic regression model with factors for treatment group and centre and a covariate for baseline throat soreness severity.

Questions on non-numeric ordinal scales were analysed using a proportional odds model using PROC LOGISTIC in SAS with factors for treatment group and centre group and a covariate for baseline throat soreness severity. Questions on numeric ordinal scales were analysed using the same ANCOVA model as the primary efficacy endpoint, or by ANCOVA with factors for treatment group and centre and covariates for the baseline throat soreness and the relevant baseline score for the specific question.

## Results

### Participant flow and baseline demographics

**Figure 1:**
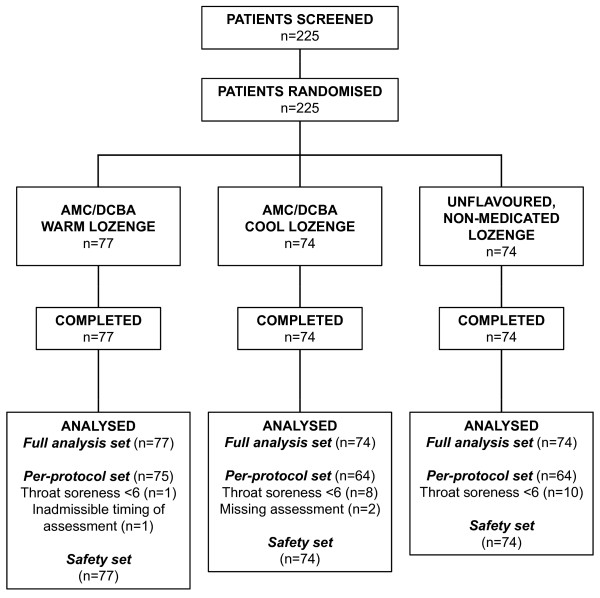
**Patient flow for selection, randomisation and analysis**.

A total of 225 patients were enrolled into the study and randomised to receive one of three study lozenges (Figure 1). No patients withdrew from the study. The intention-to-treat (ITT) and safety analyses sets were identical and consisted of all 225 patients randomised. Twenty-two patients (two from the AMC/DCBA Warm lozenge group, 10 from the AMC/DCBA Cool lozenge group and 10 from the unflavoured, non-medicated lozenge group) were excluded from the per-protocol (PP) analysis set, which therefore consisted of 203 patients, mostly owing to baseline throat soreness being too low (Figure 1). The only variables assessed with the PP analysis set were the primary efficacy endpoint and TOTPAR. Baseline demographics data show that the patients enrolled were predominantly Caucasian with slightly more women than men; the overall mean age was 31.7 years, and the mean duration of sore throat was 2.2 days. All of these parameters were well balanced between each of the treatment groups (Table 1).

**Table 1:** Patient demographics - ITT set

Variable	AMC/DCBA Warm lozenge	AMC/DCBA Cool lozenge	Unflavoured, non-medicated lozenge	Overall
Number of patients (n)	77	74	74	225
Age (year) (Mean ± SD)	30.3 ± 12.2	32.4 ± 14.7	32.6 ± 13.2	31.7 ± 13.3
Gender (% male)	41.6	39.2	41.9	40.9
Race (% Caucasian)	97.4	97.3	95.9	96.9
Alcohol drinker (%)	83.1	86.5	75.7	81.8
Current smoker (%)	36.4	37.8	35.1	36.4
Former smoker (%)	26.0	17.6	14.9	19.6
Duration of sore throat (days) (Mean ± SD)	2.3 ± 0.8	2.2 ± 0.7	2.0 ± 0.9	2.2 ± 0.8
Duration of URTI (days) (Mean ± SD)	3.0 ± 2.7	2.4 ± 1.0	3.6 ± 7.1	3.0 ± 4.4

SD, standard deviation; URTI, upper respiratory tract infection.

### Primary efficacy endpoint

#### AUC from baseline to 2 hours in severity of throat soreness

The AMC/DCBA Warm lozenge produced significantly different AUC results for the change from baseline to 2 hours in severity of throat soreness compared with the unflavoured, non-medicated lozenge (p = 0.001; Table 2). Similarly, the AMC/DCBA Cool lozenge also significantly reduced the severity of throat soreness versus the unflavoured, non-medicated lozenge, as measured by AUC for the change from baseline to 2 hours (p < 0.0001). In the ITT analysis, the terms for treatment and baseline throat soreness were both statistically significant (p < 0.0001), whereas the term for centre was not (p = 0.84). Statistical conclusions from the PP analysis were qualitatively identical to those obtained with the ITT analysis (Table 2).

**Table 2:** AUC from baseline to 2 hours post-dose for the change from baseline in throat soreness

	AMC/DCBA Warm lozenge	AMC/DCBA Cool lozenge	Unflavoured, non-medicated lozenge
**ITT Set**			
N	77	74	74
Mean ± SD	-1.83 ± 1.50	-2.07 ± 1.47	-1.00 ± 1.61
LS mean^a^	-1.78	-2.06	-0.98
Parameter estimates	LS mean^b^	95% CI	P-value
AMC/DCBA Warm lozenge - unflavoured, non­medicated lozenge	-0.80	-1.27,-0.33	0.001 **
AMC/DCBA Cool lozenge - unflavoured non­medicated lozenge	-1.08	-1.56,-0.60	<0.0001 ***
**PP Set**			
N	75	64	64
Mean ± SD	-1.87 ± 1.50	-2.16 ± 1.50	-1.25 ± 1.39
LS mean^a^	-1.83	-2.09	-1.11
Parameter estimates	LS mean^b^	95% CI	P-value
AMC/DCBA Warm lozenge - unflavoured, non­medicated lozenge	-0.72	-1.21,-0.23	0.004 **
AMC/DCBA Cool lozenge - unflavoured, non­medicated lozenge	-0.98	-1.48,-0.47	0.0002 ***

a Estimated from ANCOVA model with factors for treatment and centre and a covariate for baseline throat soreness.

b A negative difference favours the first treatment against second treatment.

** Significantly different compared with the unflavoured, non-medicated lozenge at the 1% level.

*** Significantly different compared with the unflavoured, non-medicated lozenge at the 0.1% level.

CI, confidence interval; LS, least-squares; SD, standard deviation; ITT, intention-to-treat; PP, per-protocol.

Throat soreness measured on an 11-point scale where 0 = Not sore, 10 = Very sore

### Secondary endpoints

#### Effect on severity of throat soreness

**Figure 2:**
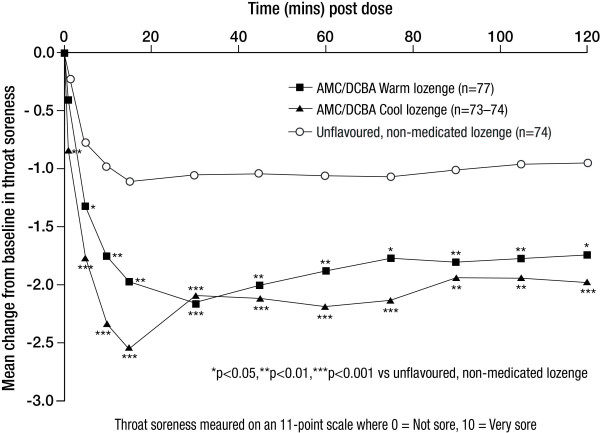
**Mean change from baseline in throat soreness from 1 to 120 minutes post dose - ITT set**.

Both AMC/DCBA Warm and AMC/DCBA Cool lozenges produced significant changes from baseline in throat soreness between 5 and 120 minutes and 1 and 120 minutes post dose, respectively, compared with the unflavoured, non-medicated lozenge (all p < 0.05, all p < 0.01, respectively; Figure 2).

#### Effect on sore throat relief

**Figure 3:**
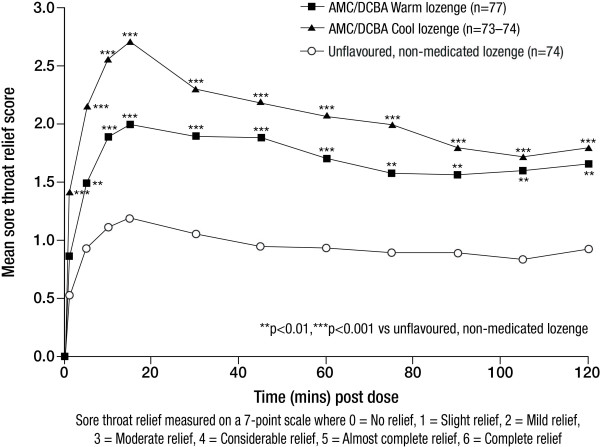
**Mean sore throat relief from 1 to 120 minutes post-dose - ITT population**.

AMC/DCBA Warm lozenges induced significant sore throat relief compared with the unflavoured, non-medicated lozenge at each assessment timepoint between 5 and 120 minutes (all p < 0.01) and AMC/DCBA Cool lozenges at each assessment timepoint between 1 and 120 minutes post dose (all p < 0.001; Figure 3). In addition, both AMC/DCBA throat lozenge variants provided significantly different TOTPAR compared with the unflavoured, non-medicated lozenge (p = 0.0001 and p < 0.0001, respectively; Table 3A).

**Table 3:** AUC data - ITT set for (A) sore throat relief (TOTPAR) from baseline to 2 hours post dose, (B) change from baseline in difficulty swallowing from baseline to 2 hours post dose and (C) throat numbness measurements from 1 to 120 minutes post dose

	AMC/DCBA Warm lozenge	AMC/DCBA Cool lozenge	Unflavoured, non-medicated lozenge
**(A) Sore throat relief (TOTPAR)**			
N	77	74	74
Mean ± SD	1.70 ± 1.19	2.06 ± 1.30	0.94 ± 1.04
LS mean^a^	1.74	2.10	0.98
Parameter estimates	LS mean^b^	95% CI	P-value
AMC/DCBA Warm lozenge - unflavoured, non­medicated lozenge	0.76	0.38,1.14	0.0001 ***
AMC/DCBA Cool lozenge - unflavoured, non­medicated lozenge	1.12	0.73,1.50	<0.0001 ***
**(B) Difficulty in swallowing**			
N	77	74	74
Mean ± SD	-13.4 ± 14.4	-19.2 ± 14.6	-7.7 ± 13.2
LS mean^c^	-13.5	-19.3	-7.5
Parameter estimates	LS mean^d^	95% CI	P-value
AMC/DCBA Warm lozenge - unflavoured, non-medicated lozenge	-5.9	-10.4,-1.5	0.009 ***
AMC/DCBA Cool lozenge - unflavoured, non-medicated lozenge	-11.7	-16.2,-7.2	<0.0001 ***
**(C) Throat numbness**			
N	77	74	74
Mean ± SD	1.86 ± 0.83	2.18 ± 0.86	1.54 ± 0.72
LS mean^a^	1.80	2.12	1.48
Parameter estimates	LS mean^b^	95% CI	P-value
AMC/DCBA Warm lozenge - unflavoured, non-medicated lozenge	0.32	0.06,0.58	0.017 *
AMC/DCBA Cool lozenge - unflavoured, non-medicated lozenge	0.64	0.38,0.90	<0.0001 ***

a Estimated from ANCOVA model with factors for treatment and centre and a covariate for baseline throat soreness.

b A positive difference favours the first treatment against second treatment.

c Estimated from ANCOVA model with factors for treatment and centre and covariates for baseline throat soreness and baseline score for difficulty in swallowing.

d A negative difference favours the first treatment against second treatment.

*** Significantly different compared with the unflavoured, non-medicated lozenge at the 0.1% level.

CI, confidence interval; LS, least-squares; SD, standard deviation.

Sore throat relief measured on a 7-point scale where 0 = No relief, 1 = Slight relief, 2 = Mild relief, 3 = Moderate relief, 4 = Considerable relief, 5 = Almost complete relief, 6 = Complete relief.

Difficulty in swallowing measured on 100 mm VAS where 0 mm = Not difficult, 100 mm = Very difficult.

Throat numbness measured on a 5-point scale where 1 = None, 2 = Mild, 3 = Moderate, 4 = Considerable, 5 = Complete.

#### Effect on difficulty in swallowing

**Figure 4:**
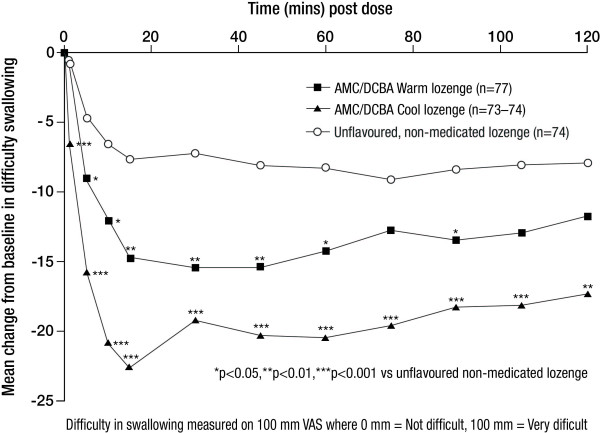
**Mean change from baseline in difficulty swallowing from 1 to 120 minutes post dose - ITT set**.

The AUC for change from baseline to 2 hours post dose in difficulty in swallowing showed significant differences with both AMC/DCBA throat lozenge variants compared with the unflavoured, non-medicated lozenge (Table 3B). At 1 minute post dose, the AMC/DCBA Cool lozenge induced significantly different changes from baseline in difficulty in swallowing compared with the unflavoured, non-medicated lozenge (p < 0.0001). A significant improvement in swallowing remained at each of the subsequent assessment timepoints between 5 and 120 minutes (all p < 0.01). The AMC/DCBA Warm lozenge induced significantly different changes from baseline in difficulty in swallowing compared with the unflavoured, non-medicated lozenge at each of the timepoints between 5 and 60 minutes and at 90 minutes post dose (all p < 0.05; Figure 4).

#### Effect on throat numbness

**Figure 5:**
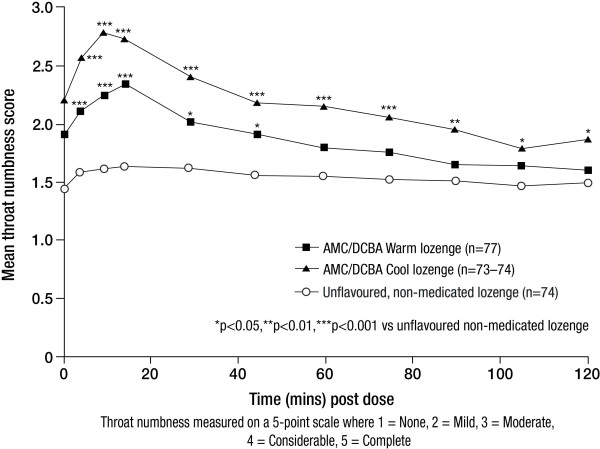
**Mean throat numbness from 1 to 120 minutes post-dose - ITT set**.

The AUC data for throat numbness measurements from 1 to 120 minutes post dose demonstrated significant differences between both AMC/DCBA throat lozenge variants and the unflavoured, non-medicated lozenge (Table 3C). Both AMC/DCBA Warm and AMC/DCBA Cool lozenges induced significant throat numbness compared with the unflavoured, non-medicated lozenge, an effect that peaked at 15 minutes post dose for AMC/DCBA Warm lozenge (p < 0.001) and 10 minutes post dose for AMC/DCBA Cool lozenge (p < 0.001; Figure 5).

#### Effect on functional impairment scores

Both AMC/DCBA Warm and AMC/DCBA Cool lozenges provided statistically significant improvements in swallowing compared with the unflavoured, non-medicated lozenge (p = 0.018 and p = 0.011, respectively; Table 4). For talking and the overall score, only the AMC/DCBA Warm lozenge achieved statistically significant reductions versus the unflavoured, non-medicated lozenge (p = 0.003 and p = 0.03, respectively). No significant difference was observed between either of the AMC/DCBA throat lozenge variants and unflavoured, non-medicated lozenge for concentrating and reading (Table 4).

**Table 4:** Change from pre-dose to 2 hours post dose in the functional impairment scale (each component and overall total score) - ITT set

	AMC/DCBA Warm lozenge	AMC/DCBA Cool lozenge	Unflavoured, non-medicated lozenge
**Talking**			
N	77	73	74
Mean ± SD	-1.09 ± 2.10	-0.56 ± 2.06	-0.20 ± 2.04
LS mean^a^	-1.49	-0.99	-0.53
Parameter estimates	LS mean^b^	95% CI	P-value
AMC/DCBA Warm lozenge - unflavoured, non­medicated lozenges	-0.96	-1.59,-0.33	0.003 **
AMC/DCBA Cool lozenge - unflavoured, non­medicated lozenges	-0.46	-1.10,0.18	0.15
**Swallowing**			
N	77	73	74
Mean ± SD	-1.35 ± 1.89	-1.36 ± 2.07	-0.65 ± 1.86
LS mean^a^	-1.51	-1.57	-0.80
Parameter estimates	LS mean^b^	95% CI	P-value
AMC/DCBA Warm lozenge - unflavoured, non­medicated lozenges	-0.71	-1.30,-0.13	0.018 *
AMC/DCBA Cool lozenge - unflavoured, non­medicated lozenges	-0.77	-1.36,-0.17	0.011 *
**Concentrating**			
N	77	73	74
Mean ± SD	-0.57 ± 1.82	-0.70 ± 1.83	-0.43 ± 1.28
LS mean^a^	-0.86	-0.82	-0.63
Parameter estimates	LS mean^b^	95% CI	P-value
AMC/DCBA Warm lozenge - unflavoured, non­medicated lozenges	-0.23	-0.71,0.25	0.34
AMC/DCBA Warm lozenge - AMC/DCBA Cool lozenge	-0.19	-0.68,0.29	0.44
**Reading**			
N	77	73	74
Mean ± SD	-0.21 ± 1.84	-0.41 ± 1.57	-0.22 ± 1.00
LS mean^a^	-0.41	-0.54	-0.36
Parameter estimates	LS mean^b^	95% CI	P-value
AMC/DCBA Warm lozenge - unflavoured, non­medicated lozenge	-0.05	-0.49,0.39	0.82
AMC/DCBA Cool lozenge - unflavoured, non­medicated lozenge	-0.18	-0.63,0.27	0.43
**TOTAL OF ALL FOUR RESPONSES**			
N	77	73	74
Mean ± SD	-3.2 ± 6.1	-3.0 ± 5.5	-1.5 ± 4.3
LS mean^a^	-4.1	-3.8	-2.3
Parameter estimates	LS mean^b^	95% CI	P-value
AMC/DCBA Warm lozenge - unflavoured, non­medicated lozenge	-1.9	-3.6,-0.2	0.03 *
AMC/DCBA Cool lozenge - unflavoured, non­medicated lozenge	-1.5	-3.2,0.1	0.07

a Estimated from ANCOVA model with factors for treatment and centre and covariates for baseline throat soreness and baseline score for the relevant variable.

b A negative difference favours the first treatment against second treatment.

* Significantly different compared with the unflavoured, non-medicated lozenge at the 5% level.

CI, confidence interval; LS, least-squares; SD, standard deviation.

Each activity measured on an 11-point scale where 0 = Would not interfere at all, 10 = Would completely interfere.

#### Early effects - Sensorial effects experienced

At the first moment of lozenge consumption, significantly more patients taking the AMC/DCBA Cool lozenge than the unflavoured, non-medicated lozenge reported experiencing soothing relief (31.1% vs 12.3% of patients, respectively; p < 0.01) and said yes to the question, 'Did the throat lozenge provide cooling relief at first moment of lozenge consumption?' (62.2% vs 9.6%, respectively; odds ratio [OR] 15.78 [95% confidence interval (CI) 6.32, 39.42]; p < 0.0001). Similarly, significantly more patients who took AMC/DCBA Warm lozenge than those who took the unflavoured, non-medicated lozenge said yes to the question, 'Did the throat lozenge provide warming relief at first moment of lozenge consumption?' (46.8% vs 13.7%, respectively; OR 5.62 [95% CI 2.50, 12.62]; p < 0.0001) - most commonly described as comforting warming, or deeply warming or gentle warming.

When asked at 1 minute, a cooling sensation was felt significantly earlier with AMC/DCBA Cool lozenge than with the unflavoured, non-medicated lozenge (OR 20.10 [95% CI 9.69, 41.69]; p < 0.0001), with 60.8% of patients experiencing this within 5 seconds and 78.4% within 10 seconds. For AMC/DCBA Warm lozenge, a warming sensation was felt significantly earlier than the unflavoured, non-medicated lozenge (OR 8.80 [95% CI 4.70, 16.49]; p < 0.0001) with 62.4% experiencing this within 30 seconds.

#### Effects at 2 hours - Duration of sensorial effects experienced

When asked at 2 hours post dose, 'How long did the cooling sensation of the throat lozenge last in your mouth?', only the AMC/DCBA Cool lozenge produced a cooling sensation in the throat that lasted significantly longer than the unflavoured, non-medicated lozenge (OR 0.11 [95% CI 0.06, 0.21]; p < 0.0001).

Similarly, when the same question was asked at 2 hours about the duration of warming sensation, only the AMC/DCBA Warm lozenge produced a warming sensation in the throat that lasted significantly longer than the unflavoured, non-medicated lozenge (OR 0.16 [95% CI 0.09, 0.30]; p < 0.0001).

#### Emotional benefits

Significantly more patients who received one of the two AMC/DCBA throat lozenge variants (51.9%, 57.5%, for AMC/DCBA Warm and AMC/DCBA Cool lozenge, respectively) said yes to the question, 'At 2 hours post dose, do you feel any better than before you took the throat lozenge?' compared with those who received the unflavoured, non-medicated lozenge (19.2%; both p < 0.0001).

**Figure 6:**
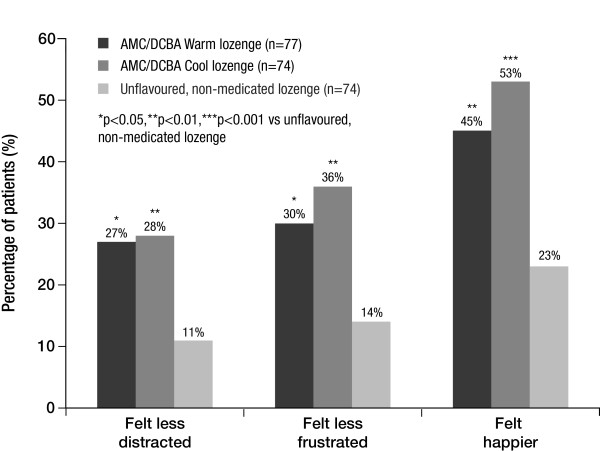
**Proportion of patients who said they felt less distracted, less frustrated and happier after taking their pre-assigned throat lozenge**.

In addition, significantly more patients who took either one of the AMC/DCBA throat lozenge variants reported feeling less distracted, less frustrated and happier than before they took the throat lozenge at 2 hours post dose compared with those who took an unflavoured, non-medicated lozenge (p < 0.02 in all cases; Figure 6).

#### Overall ratings on throat lozenge as a treatment for sore throat

Both AMC/DCBA throat lozenge variants yielded significantly higher scores than unflavoured, non-medicated lozenge (p < 0.0001 in both cases) when the question, 'How would you rate this throat lozenge as a treatment for sore throat?' was asked at 2 hours and recorded on an 11-point scale where 0 = Poor and 10 = Excellent. The least squares (LS) mean difference between the AMC/DCBA Warm lozenge, AMC/DCBA Cool lozenge and the unflavoured, non-medicated lozenge was 2.57 (95% CI 1.68, 3.45) and 3.00 (95% CI 2.11, 3.90), respectively; both p < 0.0001.

### Adverse events

Eighteen out of 225 patients (8%) reported a total of 23 treatment-emergent adverse events. The highest number of events was reported by patients in the unflavoured, non-medicated lozenge group with 10 patients reporting 11 treatment-emergent events, while four patients in the AMC/DCBA Warm lozenge group reported a total of eight events and four patients in the AMC/DCBA Cool lozenge group reported one event each. The majority of events (20 out of 23, i.e. 87%) were mild in severity, and none were considered to be definitely, probably or possibly related to the study medication. There were no treatment-emergent serious adverse events.

Most adverse events were related to the patient's URTI, such as headache, cough and congestion, with headache being the most common treatment-emergent adverse event reported (i.e. seven reports by seven patients; three from the unflavoured, non-medicated lozenge group and two from each of the AMC/DCBA throat lozenge variant groups). There were no statistically significant pair-wise treatment differences between the treatment groups in the proportion of patients reporting treatment-emergent adverse events.

## Discussion

The results obtained were robust with qualitatively identical conclusions drawn from the equivalent PP analyses where performed. In addition, there was no evidence to suggest that the results differed significantly between centres. A single dose of the AMC/DCBA Warm lozenge and the AMC/DCBA Cool lozenge exhibited significant analgesic, functional and emotional benefits compared with the unflavoured, non-medicated lozenge, and also demonstrated a number of sensorial effects. With the AMC/DCBA Warm lozenge, sore throat relief and improvements in throat soreness and difficulty in swallowing were observed as early as 5 minutes post dose, and lasted for up to 2 hours. Similarly, the AMC/DCBA Cool lozenge provided sore throat relief and improvements in throat soreness and difficulty in swallowing, but at the earlier timepoint of 1 minute post dose, which also lasted long after the lozenge had dissolved, for up to 2 hours post dose. The mean time (± standard deviation) for a AMC/DCBA throat lozenge to dissolve in the mouth was previously investigated and found to be 6.77 ± 2.01 min [17].

Both of the AMC/DCBA throat lozenge variants induced throat numbness from 1 minute post dose and were significantly different from the unflavoured, non-medicated lozenge for the duration of 2 hours, as indicated by the AUC data. This can be explained by the findings of the study by Buchholz *et al*. [11] where the two active ingredients of AMC/DCBA throat lozenges, AMC and DCBA, were found to act in a local anaesthetic-like manner by blocking voltage-gated neuronal sodium channels in a similar way to lidocaine [11]. The peak effects observed in this single dose study for pain relief, throat soreness, difficulty in swallowing and throat numbness were achieved by 15 and 30 minutes for the AMC/DCBA Cool and AMC/DCBA Warm lozenge after initial dosing and lasted for up to 2 hours, suggesting that the relief provided by both AMC/DCBA throat lozenge variants is not confined to the time the throat lozenge remains in the mouth, and that relief is felt long after the throat lozenge has dissolved. Significant analgesic and functional effects on severity of throat soreness, sore throat relief and difficulty in swallowing have previously been demonstrated with the original AMC/DCBA throat lozenge (which contains only AMC and DCBA) compared with an unflavoured, non-medicated lozenge, the benefits of which were rapid in onset and ongoing, lasting long after the lozenge had gone [15].

Unsurprisingly, for patients with a sore throat the two functional areas that were considered to be most impaired at baseline were swallowing and talking, which support what is currently known [15]. Moreover, the analgesic benefits reported by the patients translated into functional benefits, with differences in favour of the AMC/DCBA Cool and AMC/DCBA Warm lozenge for swallowing, and in the case of the AMC/DCBA Warm lozenge, for talking also, compared with the unflavoured, non-medicated lozenge. In addition, patients taking either one of the AMC/DCBA throat lozenge variants reported experiencing soreness relief, relief from burning, soothing relief, warming relief and pain relief from the moment they took the throat lozenge, supporting the instant effects of these lozenges.

The consumer questionnaire also qualified the sensorial benefits associated with each of the variants over and above the efficacy benefits observed, i.e. AMC/DCBA Warm lozenge was associated with a comforting, warming sensation and AMC/DCBA Cool lozenge was associated with a cooling sensation. These sensorial effects were felt instantly as soon as the lozenge was sucked and in the case of the AMC/DCBA Cool lozenge, the cooling sensation lasted for up to 2 hours.

Furthermore, when asked at 2 hours, patients who took either one of the AMC/DCBA throat lozenge variants reported feeling better, less distracted, less frustrated and happier than before they took the lozenge - all of which are emotional benefits. Thus, the results of this study suggest that the patients were able to feel more like themselves and continue with their everyday lives after taking either of the two AMC/DCBA throat lozenge variants. It is not unexpected that patients taking these lozenges would experience both emotional and physical benefits of pain relief as demonstrated in this study because if patients are feeling less pain, they are more likely to be feeling better. As previously demonstrated, sore throat and 'sickness' are highly correlated with each other, where a greater level of pain experienced by the patient is associated with a greater feeling of sickness/unwellness, and vice versa [3].

Other analgesic studies have concluded that a reduction of 1-2 points on an 11-point ordinal scale represented clinically important differences [18-21]. The magnitude of the changes observed in the present study both in terms of changes from baseline of each of the efficacy endpoints, and between each of the AMC/DCBA throat lozenge variants compared with the unflavoured, non-medicated lozenge are therefore clinically meaningful. These clinically meaningful observations are in keeping with those reported for the original AMC/DCBA throat lozenge [15]. Also in keeping with previously published data is the 'placebo effect' observed with the unflavoured, non-medicated lozenge [15]. However, this study only investigated the effects of the two AMC/DCBA throat lozenge variants as a whole in patients with sore throat, and did not investigate the sensory impact of the excipients of the two lozenge formulation variants on the placebo effect, which warrants further investigation.

Similar to the findings from two surveys in healthy volunteers, the sensorial benefits of a cooling sensation and a warming sensation experienced by healthy adults who took a AMC/DCBA Cool or a AMC/DCBA Warm lozenge, respectively, were mirrored in patients with acute sore throat, and furthermore, were translated into both analgesic and functional benefits (Personal Communications) [16].

## Conclusions

In conclusion, AMC/DCBA Warm lozenges and AMC/DCBA Cool lozenges are well-tolerated and effective OTC treatment options, offering not only functional, but also instant sensorial as well as emotional benefits to patients with acute sore throat, over and above that of the rapid clinically meaningful improvements in efficacy that ensued.

## Competing interests

AGW and GMC are directors of CPS Research who have received research grants from Reckitt Benckiser Healthcare International for the conduct of this and previous research.

CM and AS are employees of Reckitt Benckiser Healthcare International, who are the manufacturers of Strepsils. MAG is a Senior Statisician at Worldwide Clinical Trials, contracted by Reckitt Benckiser Healthcare International for this research. This study was funded by Reckitt Benckiser Healthcare International, UK.

## Authors' contributions

AGW was involved in the design of the study, the recruitment and management of the patients, the interpretation of the results and detailed review of the manuscript. GMC was involved in the study design, co-ordinated recruitment and management of patients and reviewed the manuscript. CM and AS were involved in the study design, interpretation of results and review of the manuscript. MG was the contract statistician involved in the statistical/data analyses of this study. All authors have read, reviewed and approved the manuscript.

## Pre-publication history

The pre-publication history for this paper can be accessed here:

http://www.biomedcentral.com/1471-2296/12/6/prepub
